# Feasibility of utilizing mediastinal drains alone following esophageal cancer surgery: a retrospective study

**DOI:** 10.1186/s12957-024-03400-x

**Published:** 2024-05-03

**Authors:** Yu Li, Danjie Zhang, Danwen Zhao

**Affiliations:** Department of Thoracic Surgery, The Second Affiliated Hospital of Xi’an Jiaotong University, No. 157#, The West 5th Road, Xi’an, 710004 Shaanxi China

**Keywords:** Esophageal cancer, Mediastinal drainage, Pain score, Anastomotic leakage

## Abstract

**Background:**

It was typically necessary to place a closed thoracic drainage tube for drainage following esophageal cancer surgery. Recently, the extra use of thoracic mediastinal drainage after esophageal cancer surgery had also become more common. However, it had not yet been determined whether mediastinal drains could be used alone following esophageal cancer surgery.

**Methods:**

A total of 134 patients who underwent esophageal cancer surgery in our department between June 2020 and June 2023 were retrospectively analyzed. Among them, 34 patients received closed thoracic drainage (CTD), 58 patients received closed thoracic drainage combined with mediastinal drainage (CTD-MD), while 42 patients received postoperative mediastinal drainage (MD). The general condition, incidence of postoperative pulmonary complications, postoperative NRS score, and postoperative anastomotic leakage were compared. The Mann-Whitney *U* tests, Welch’s *t* tests, one-way ANOVA, chi-square tests and Fisher’s exact tests were applied.

**Results:**

There was no significant difference in the incidence of postoperative hyperthermia, peak leukocytes, total drainage, hospitalization days and postoperative pulmonary complications between MD group and the other two groups. Interestingly, patients in the MD group experienced significantly lower postoperative pain compared to the other two groups. Additionally, abnormal postoperative drainage fluid could be detected early in this group. Furthermore, there was no significant change in the incidence of postoperative anastomotic leakage and the mortality rate of patients after the occurrence of anastomotic leakage in the MD group compared with the other two groups.

**Conclusions:**

Using mediastinal drain alone following esophageal cancer surgery was equally safe. Furthermore, it could substantially decrease postoperative pain, potentially replacing the closed thoracic drain in clinical practice.

## Introduction

Esophageal cancer was a widespread malignant tumor globally, with 604,100 new cases reported worldwide in 2020, ranking eighth in the incidence of malignant tumors globally [1]. China had a high incidence of esophageal cancer, accounting for over half of the new cases worldwide [2]. China had 324,000 new cases of esophageal cancer in 2020, ranking it sixth for malignant tumor incidence in the country [3]. Currently, the treatment for early or locally advanced esophageal cancer primarily involved surgery, along with radiotherapy, chemotherapy, and immunotherapy [2, 4].

Esophageal cancer surgeries mainly included open and minimally invasive procedures. Almost all postoperative esophageal cancer patients required drainage tubes in the thoracic cavity to promote the drainage of pleural effusion and pneumatoconiotics [5, 6]. For a long time, 1–2 closed thoracic drains had been typically placed after esophageal cancer surgery to facilitate thoracic drainage [7]. In recent years, the placement of thoracic mediastinal drains after esophageal cancer surgery had also become more common. The thoracic mediastinal drains varied in diameter from 14 to 26 Fr and were placed near the anastomosis in the esophageal bed [8–10]. It was typically used in conjunction with a closed thoracic drain to ensure sufficient postoperative drainage [11]. Studies had shown that placing a thoracic mediastinal drain after esophageal cancer surgery help in the early diagnosis and healing of esophageal anastomotic fistula, and promote the recovery of patients [8, 11]. One study had shown that using a single mediastinal drain through the abdominal wall after esophagectomy may be feasible [10]. However, there remains no conclusive evidence on whether thoracic mediastinal drains could be used alone in postoperative patients with esophageal cancer. Therefore, we retrospectively analyzed the data of 134 patients who underwent esophageal cancer surgery in our department between June 2020 and June 2023 to investigate the feasibility of using thoracic mediastinal drains alone after the surgery.

## Methods

### Patients

This study included 134 patients with esophageal cancer who underwent esophageal cancer surgery from June 2020 to June 2023 in the Department of Thoracic Surgery of the Second Affiliated Hospital of Xi’an Jiaotong University. The inclusion criteria of patients were as follows: (1) age ≤ 80 years; (2) pathological diagnosis of esophageal cancer; (3) no abnormality in heart, lung and brain; (4) no distant metastasis of the tumor. The exclusion criteria for patients were as follows: (1) severe heart, brain, and lung disease; (2) severe thoracic adhesions or history of previous thoracic surgery. All patients underwent routine preoperative tests before surgery, including blood routine, liver function, lung ventilation function, and chest CT. The study was approved by the Ethics Committee of Xi’an Jiaotong University College of Medicine according to the principles outlined in the Declaration of Helsinki.

### Surgery and drainage methods

**Fig. 1:**
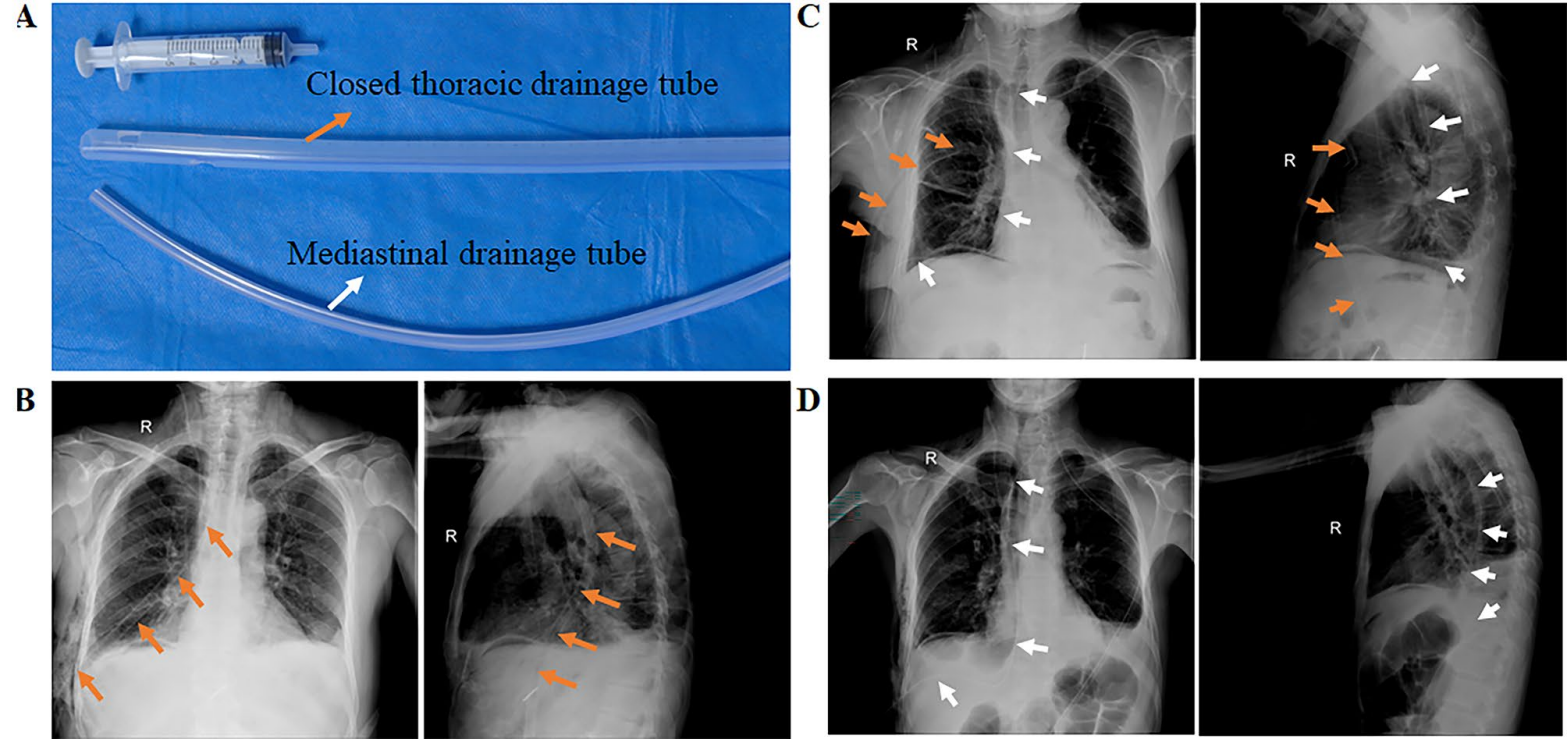
(**A**) Closed thoracic drainage tube and mediastinal drainage tube; (**B**-**D**) Placement of closed thoracic drainage tube and mediastinal drainage tube in the three groups. CTD: Closed thoracic drainage; MD: Mediastinal drainage. Orange arrows represent the position of the CTD and white arrows represent the position of the MD.

Part of the patients underwent preoperative neoadjuvant therapy according to NCCN guidelines [12]. All patients underwent surgery performed by experienced surgeons in accordance with NCCN clinical practice guidelines. We performed various esophageal cancer surgeries, including McKeown esophagectomy, Ivor Lewis esophagectomy, and Sweet esophagectomy. The surgeon completely freed the esophagus and dissected the lymph nodes based on the tumor’s location. Subsequently, the surgeon completely freed the stomach and shaped it into a tubular form, then pulled it into the pleural cavity or neck for anastomosis. Of these, the anastomosis for the McKeown esophagectomy was located in the neck, and the anastomosis for the Ivor Lewis esophagectomy and Sweet esophagectomy was located in the pleural cavity [13–15]. In the CTD group, a 36-Fr closed thoracic drainage tube was inserted through the chest wall at the intersection of the seventh intercostal space and the mid-axillary line, reaching the apex of the pleural cavity. The other end of the tube was connected to a water-seal bottle (Fig. 1B). In the MD group, a 19- French mediastinal drainage tube was placed posterior to the anastomosis, passed through the mediastinal esophageal bed and the thoracic cavity to the level of the diaphragm, and secured to the chest wall at the intersection of the seventh intercostal space and the anterior axillary line (Fig. 1D). According to the location of the anastomosis, the implantation distance of the mediastinal drain varies from 20 to 28 cm. A one-way valve ball was connected to the end of the mediastinal drain to create negative pressure. In the CTD-MD group, we used two drains simultaneously (Fig. 1C). Both left-sided approach and right-sided approach were included in each group of included cases. The representative images in Fig. 1 had been shown with a right-sided approach as an example. After the surgery, the anesthesiologist reinflated the lungs while directly observing them and confirmed that they were fully re-expanded.

### Postoperative management

All patients received appropriate parenteral nutrition from the first to the third postoperative days. Adequate enteral nutrition was provided through an enteral nutrition tube placed intraoperatively starting from the fourth day after the operation, in the absence of any special circumstances. All patients underwent blood routine, liver function, kidney function, and electrolytes tested on the first postoperative day and then repeated every three days. Our nursing team routinely monitored the patient’s axillary temperature and drainage flow on a daily basis. All patients underwent chest CT scans and esophagography (swallowed contrast examination) successively within 7 days after surgery. The drains were removed once the following criteria were met simultaneously: drainage of less than 150 ml per drain per day, absence of abnormalities in the drainage fluid, no air bubbles overflowing in the water-sealed bottle or negative pressure suction device maintaining negative pressure, and no anastomotic leakage or significant pulmonary complications. For the protocol of pain control, all patients received intravenous flurbiprofen acetate (100 mg twice daily, postoperative days 1–4), following the standard practice at our institution, without performing a paravertebral block.

### Outcome measures

Hyperthermia was defined as a patient’s temperature exceeding 39.0 °C. The peak leukocytes referred to the highest value of leukocytes in the patient’s postoperative blood routine. The total drainage volume was the sum of the daily drainage volume from the time the drain was placed to the time it was removed. Hospitalization days referred to the total number of days a patient was hospitalized from admission to discharge.

### Major pulmonary complications

The distance between the edge of the lung and the chest wall on a CT chest scan was greater than 3 centimeters, indicating atelectasis. Pleural effusion was defined as a chest CT showing evidence of pleural effusion with lung compression exceeding 30%. Severe pulmonary infection was defined as the presence of infiltrates in at least one lobe on chest CT, accompanied by significant chest tightness and shortness of breath.

### Numerical rating scale (NRS)

Currently, the numerical rating scale (NRS) was the most common measurement instrument for assessing postoperative pain in adults [16]. All surgical patients received health education upon admission, which included information on pain assessment methods. All patients were assessed three times a day at 7 a.m., 3 p.m., and 11 p.m. The average pain score for the day was calculated and continued to be assessed for four days after the operation.

### Anastomotic leakage

Anastomotic leakage (AL) was defined as a full-thickness gastrointestinal defect involving the esophagus, anastomosis, staple line, or conduit, regardless of presentation or method of identification [17]. In the study, we investigated the occurrence of anastomotic leakage by using a combination of postoperative esophagography, e-gastroscopy, and chest CT scans of the patients. In addition, a sudden increase in drainage fluid, turbidity, and foul odor were considered abnormal. The time of the first occurrence of abnormal drainage in AL patients was recorded.

### Statistical analysis

Categorical variables were presented as frequencies and percentages. Differences in categorical variables were analyzed using chi-squared tests or Fisher’s exact tests (SPSS 20.0, Chicago, IL, USA). Continuous variables were presented as mean ± standard deviation (SD) and median (interquartile range, IQR). The Mann-Whitney *U* tests were used in cases of nonnormal data distribution (SPSS 20.0, Chicago, IL, USA). For normal data distribution, continuous variables of two groups were examined by the Levene test. When variances were not equal, the Welch’s *t* test was performed, and when variances were equal, one-way ANOVA was employed (SPSS 20.0, Chicago, IL, USA). Differences were considered significant as *p* < 0.05.

## Results

### Demographic and clinical characteristics

A total of 134 patients who underwent surgery for esophageal cancer were included in this study. Among them, 34 patients underwent closed thoracic drainage (CTD) following surgery, 58 patients underwent closed thoracic drainage combined with mediastinal drainage (CTD-MD) following surgery, and 42 patients underwent postoperative mediastinal drainage (MD) following surgery. The demographic data and clinical characteristics of all patients were presented in Table 1. There were no statistical differences among the three groups of patients in terms of age, gender, smoking status, tumor location, pathology type, neoadjuvant therapy status, preoperative maximal ventilation in percentage of predicted value (MVV%), preoperative total protein, preoperative hemoglobin (Hb), surgical procedure, surgery duration and intraoperative infusion volume (Table 1).

**Table 1:** Demographic and clinical characteristics

Variable	CTD (*n* = 34)	CTD-MD (*n* = 58)	MD (*n* = 42)	*p*
**Age (years)**				0.62
Mean ± SD	63.71 ± 8.26	63.14 ± 9.57	64.76 ± 5.64	
Median (IQR^a^)	63.00 (59.50, 69.00)	63.00 (56.00, 70.50)	66.50 (62.25, 69.00)	
**Sex**				0.75
Female	7 (20.59%)	16 (27.59%)	10 (23.81%)	
Male	27 (79.41%)	42 (72.41%)	32 (76.19%)	
**Smoking**				0.80
No	14 (41.17%)	28 (48.27%)	19 (45.23%)	
Yes	20 (58.83%)	30 (51.73%)	23 (54.77%)	
**Tumor location**				0.87
Proximal	5 (14.71%)	11 (18.97%)	9 (21.42%)	
Distal	17 (50.00%)	26 (44.82%)	16 (38.10%)	
Mid	12 (35.29%)	21 (36.21%)	17 (40.48%)	
**Pathology type**				0.62
Squamous carcinoma	25 (73.53%)	35 (60.34%)	24 (57.14%)	
Adenocarcinoma	6 (17.65%)	13 (22.42%)	11 (26.19%)	
Others	3 (8.82%)	10 (17.24%)	7 (16.67%)	
**Neoadjuvant therapy**				0.89
No	15 (44%)	22 (38%)	20 (48%)	
Chemotherapy	13 (38%)	26 (45%)	15 (36%)	
Chemoradiotherapy	6 (18%)	10 (17%)	7 (17%)	
**Preoperative MVV%**				0.99
Mean ± SD	93.29 ± 12.31	93.36 ± 11.98	93.10 ± 9.44	
Median (IQR)	93.50 (84.25, 97.00)	88.50 (85.00, 102.00)	91.50 (86.25, 97.75)	
**Preoperative total protein ≥ 65 g/L**				0.85
No	21 (62%)	33 (57%)	26 (62%)	
Yes	13 (38%)	25 (43%)	16 (38%)	
**Preoperative Hb ≥ 13 g/dL**				0.50
No	20 (59%)	37 (64%)	30 (71%)	
Yes	14 (41%)	21 (36%)	12 (29%)	
**Surgical procedure**				0.89
Ivor Lewis	15 (44.12%)	21 (36.20%)	15 (35.71%)	
McKeown	6 (17.64%)	14 (24.14%)	11 (26.19%)	
Sweet	13 (38.24%)	23 (39.66%)	16 (38.10%)	
**Surgery time (min)**				0.74
Mean ± SD	222.18 ± 78.89	227.03 ± 78.06	236.21 ± 89.71	
Median (IQR)	222.50 (157.50, 256.00)	235.00 (163.50, 263.00)	235.50 (152.00, 291.25)	
**Intraoperative infusion volume (ml)**				0.14
Mean ± SD	3,339.65 ± 345.77	3,212.97 ± 435.28	3,139.50 ± 503.11	
Median (IQR)	3,285.00 (3,015.25, 3,543.75)	3,272.50 (2,875.25, 3,542.75)	3,243.50 (2,781.25, 3,445.25)	

^a^IQR: Interquartile Range

### Mediastinal drain alone may not affect postoperative recovery in patients with esophageal cancer

In order to understand the impact of different drainage measures on postoperative recovery, we gathered data on the incidence of postoperative hyperthermia, peak leukocytes, total drainage volume, and hospitalization days. The results indicated that there was no statistical difference in the incidence of postoperative hyperthermia among the three groups of patients (Table 2). There was also no significant difference in the peak leukocytes in the postoperative blood routine of the three groups of patients (Table 2). In addition, there were no significant differences in the total drainage volume and hospitalization days among the three groups (Table 2). Our findings indicated that mediastinal drain alone may not affect postoperative recovery in patients with esophageal cancer.

**Table 2:** Comparison of postoperative recovery in three groups

	CTD (*n* = 34)	CTD-MD (*n* = 58)	MD (*n* = 42)	*p*
**Postoperative hyperthermia**				0.82
No	27 (79.41%)	43 (74.14%)	31 (73.81%)	
Yes	7 (20.59%)	15 (25.86%)	11 (26.19%)	
**Peak leukocytes (10** ^**9**^ **/L)**				0.75
Mean ± SD	13.59 ± 3.80	13.12 ± 2.25	13.40 ± 3.16	
Median (IQR)	12.00 (11.00, 14.75)	13.00 (12.00, 13.00)	13.00 (12.00, 13.00)	
**Total drainage volume (ml)**				0.59
Mean ± SD	2,076.47 ± 241.29	2,041.38 ± 224.03	2,083.33 ± 191.17	
Median (IQR^a^)	2,100.00 (1,900.00, 2,200.00)	2,100.00 (1,900.00, 2,200.00)	2,100.00 (1,900.00, 2,200.00)	
**Hospitalization days**				0.92
Mean ± SD	14.62 ± 2.594	14.57 ± 2.507	14.79 ± 2.850	
Median (IQR)	14.00 (13.00, 15.00)	14.00 (14.00, 16.00)	14.00 (13.00, 16.00)	

^a^IQR: Interquartile Range

### Mediastinal drain alone may not affect the incidence of pulmonary complications in postoperative patients with esophageal cancer

There was no statistically significant difference in the incidence of postoperative pulmonary atelectasis among the three groups (Table 3). The occurrence of postoperative pleural effusion did not show a statistically significant difference among the three groups of patients (Table 3). Additionally, there was no statistically significant difference in the incidence of postoperative lung infection among the three groups (Table 3). Our results suggest that mediastinal drain alone may not affect the incidence of pulmonary complications in postoperative patients with esophageal cancer.

**Table 3:** Comparison of postoperative pulmonary complications in three groups

	CTD *n* = 34	CTD-MD *n* = 58	MD *n* = 42	*p*
**Pulmonary atelectasis**				0.99
No	32 (94.12%)	54 (93.10%)	39 (92.86%)	
Yes	2 (5.88%)	4 (6.90%)	3 (7.14%)	
**Pleural effusion**				0.83
No	31 (91.18%)	54 (93.10%)	40 (95.24%)	
Yes	3 (8.82%)	4 (6.90%)	2 (4.76%)	
**Lung infection**				0.65
No	33 (97.06%)	53 (91.38%)	40 (95.24%)	
Yes	1 (2.94%)	5 (8.62%)	2 (4.76%)	

### Mediastinal drain alone could significantly relieve postoperative pain in patients with esophageal cancer

**Fig. 2:**
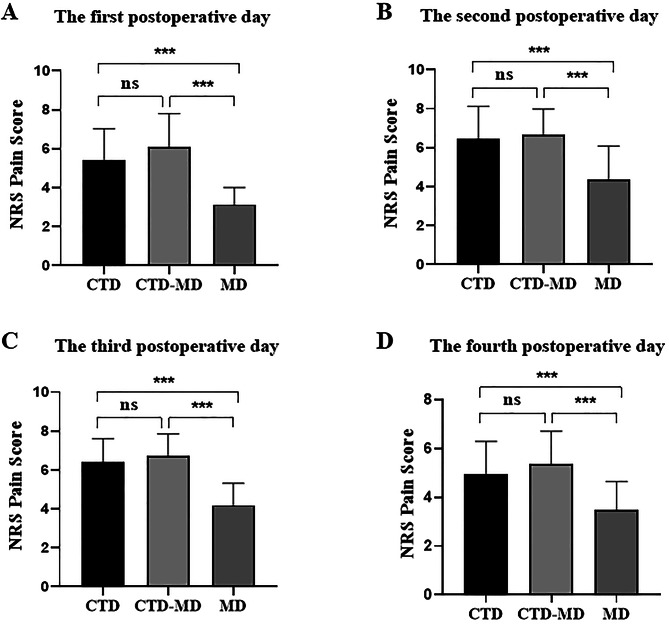
**Mediastinal drain alone could significantly relieve postoperative pain in patients with esophageal cancer.** NRS scores were assessed on postoperative days 1 (**A**), 2 (**B**), 3 (**C**), and 4 (**D**) in each group. Statistical analysis was performed by Student *t*-test. Values are presented as mean ± SD. *p < 0.05; **p < 0.01; ***p < 0.001

Our findings revealed that on postoperative days 1st, 2nd, 3rd, and 4th, the NRS scores of patients in the MD group were significantly lower than those of the CTD group and CTD-MD groups (Fig. 2). There were no statistically significant differences between the CTD group and CTD-MD group (Fig. 2). Hence, a mediastinal drain alone could significantly alleviate postoperative pain in patients with esophageal cancer.

### Mediastinal drains alone may not affect the incidence or mortality of anastomotic leaks after esophageal cancer surgery

**Table 4:** Comparison of postoperative anastomotic leakage in three groups

	CTD, *n* = 34	CTD-MD, *n* = 58	MD, *n* = 42	*p*
**AL** ^**a**^				0.80
No	30 (88.24%)	53 (91.38%)	39 (92.86%)	
Yes	4 (11.76%)	5 (8.62%)	3 (7.14%)	
**30-days patient mortality with AL**				0.76
No	2 (50.00%)	4 (80.00%)	2 (66.67%)	
Yes	2 (50.00%)	1 (20.00%)	1 (33.33%)	
**Time to first abnormal drainage fluid in patients with AL(Days)**				0.012
Mean ± SD	6.00 ± 0.82	3.40 ± 0.55	2.66 ± 0.58	
Median (IQR^b^)	6 (6, 6)	3 (3, 4)	3 (3, 3)	

^a^AL: Anastomotic leakage; ^b^IQR: Interquartile Range

Our study found that there was no significant difference in the occurrence of postoperative esophageal anastomotic leakage among the three groups of patients (Table 4). Among patients with anastomotic leakage in each group, there was also no significant difference in 30-days patient mortality (Table 4). In patients with anastomotic leakage, abnormal drainage fluid was detected earlier in both the CTD-MD and MD groups compared to the CTD group (Table 4). However, there was no significant difference between the CTD-MD group and MD group (Table 4). Our results suggest that using mediastinal drains alone may not affect the incidence or mortality of anastomotic leaks after esophageal cancer surgery. In addition, the use of mediastinal drains alone after esophageal cancer surgery may be potentially valuable for early detection of anastomotic leaks.

## Discussion

Our study found that using a thoracic mediastinal drain alone after esophageal cancer surgery is feasible. The use of a mediastinal drain alone after esophageal cancer surgery significantly reduced patients’ postoperative pain and may be potentially valuable for early detection of anastomotic leaks. In addition, the use of mediastinal drainage alone after esophageal cancer surgery showed no difference in the incidence of postoperative hyperthermia, peak leukocytes, total drainage volume, hospitalization days, incidence of postoperative pulmonary complications, incidence of postoperative anastomotic leakage, and mortality rate of patients with anastomotic leakage when compared with the traditional drainage method.

The literature reported that fever, white blood cell counts, total drainage, and hospitalization days of patients following thoracic surgery could roughly reflect their postoperative recovery [18]. Moreover, these presentations were closely related to the patient’s postoperative pulmonary complications, such as pulmonary atelectasis, pleural effusion, and pulmonary infections [19, 20]. Our study indicated that there was no significant difference between the MD group and the other two groups in the incidence of postoperative hyperthermia, peak leukocytes, total drainage volume, and hospitalization days. This suggest that using a mediastinal drain alone after esophageal cancer surgery may not impact the patient’s postoperative recovery. This finding aligned with literatures which reported that there was no significant correlation between the postoperative recovery of thoracic surgery patients and the number of drainage tubes [21–23]. However, there was also the view that the placement of multiple drains after thoracic surgery will result in smoother drainage to ensure adequate air and fluid drainage, which was more favorable to the patient’s postoperative recovery [24, 25]. In this study, the anesthesiologist re-inflated the lungs under direct observation after surgery to confirm that the lungs had been completely re-expanded. And this study has excluded patients with severe thoracic adhesions. Therefore, the probability of a postoperative air leak in the included patients was low. The main problem was the drainage of pleural fluid. We utilized a mediastinal drain with drainage slots at the tip and various parts of the tip to ensure adequate drainage of fluid from the anastomosis to the bottom of the chest cavity, which is equivalent to closed thoracic drainage. Therefore, the use of mediastinal drains alone may be equally safe.

Pulmonary complications were the relatively common postoperative outcomes of esophageal cancer, and they had a detrimental effect on the survival of patients [26, 27]. It had been suggested that pulmonary complications may independently predict a worse prognosis in postoperative patients with esophageal cancer [28]. The most common pulmonary complications following esophageal cancer surgery included pleural effusion, pulmonary atelectasis, and lung infection [29]. In this study, we found no significant difference in the incidence of postoperative pleural effusion, pulmonary atelectasis, and pulmonary infection in the MD group compared with the other two groups. This suggest that using only a mediastinal drain after surgery for esophageal cancer may not increase the incidence of postoperative pulmonary complications and is equally safe. This was consistent with most of the research [10]. However, some thoracic surgeons had suggested that two tubes should still be used when a high incidence of intraoperative bleeding was expected [22].

Postoperative pain following thoracic surgery was quite common. Despite improvements in recent years, attributed to the advancement of minimally invasive techniques and analgesic methods, it was important not to overlook patients’ postoperative pain [30, 31]. Postoperative pain not only affected patients’ comfort after surgery but also directly impacted their ability to cough and get out of bed early, thereby increasing the incidence of respiratory and cardiovascular complications and affecting patients’ prognosis [7, 16]. A study suggest that postoperative pain following thoracic surgery may primarily result from irritation caused by closed chest drains [32]. This finding was consistent with our results, indicating that placement of a mediastinal drain alone could significantly reduce the patient’s postoperative pain compared to the CTD and CTD-MD groups. We analyzed that the mediastinal drain may be attributed to the soft texture and thin diameter of the tube, which reduced its stimulation to the intercostal nerve, thereby potentially reducing the pain and radiating pain caused by the intercostal nerve. In addition, mediastinal drains also caused less irritation to the lungs and the pleura of the visceral layers, leading to reduced pain associated with visceral involvement. Therefore, using mediastinal drains alone after esophageal cancer surgery may have significant advantages in reducing patients’ postoperative pain. However, recent studies had shown that using a transhiatal mediastinal drainage through a laparoscopic port site may further reduce postoperative pain in patients, compared to transthoracic mediastinal drainage [33, 34]. The approach may have avoided the irritation of the drain tube on the nerves of the chest wall. In the future, we plan to make an effort in that direction to further reduce the incidence of postoperative pain in esophageal cancer.

Anastomotic leakage was one of the most serious postoperative complications of esophageal cancer, with an incidence of approximately 7.2–21.2% and a lethality rate of 7.2–35% [17, 35, 36]. Patients with severe anastomotic leakage had an extremely poor prognosis [37]. Several studies had reported that various methods of chest drainage following esophageal cancer surgery did not affect the incidence of anastomotic leakage [8–11, 38]. This finding was consistent with our study, which showed that the incidence of postoperative anastomotic leakage in patients in the MD group was not statistically different from the other two groups. Furthermore, we discovered that the utilization of mediastinal drains did not impact the mortality rate of patients experiencing anastomotic leakage following esophageal cancer surgery, aligning with the findings of Hainong Ma’s study [8]. However, it had been reported that the use of mediastinal drains after esophageal cancer surgery significantly reduces mortality due to anastomotic leakage [11]. Yin Li reported that the mortality rate of 108 patients with esophageal cancer, all of whom used mediastinal drains after surgery, was 0% [9]. The reason for this different may be due to the relatively small sample size of our study. Therefore, we may still need to increase the number of patients in later stages to validate the aforementioned results. Abnormal drainage fluid was typically one of the initial clinical signs of an anastomotic leakage, characterized by a sudden increase in the volume of drainage, as well as changes in color and odor [8, 39]. It had been found that adding a mediastinal drain to a closed chest drain after esophageal cancer surgery increases the likelihood of detecting abnormal drainage fluid at an early stage. This was beneficial for the early diagnosis of anastomotic leakage [8]. This was consistent with our findings. We also discovered that patients in the MD group with anastomotic leakage could be identified early by abnormal drainage fluid, with no statistically significant difference compared to the CTD-MD group. This suggest that using mediastinal drains alone may be potentially valuable for early detection of anastomotic leaks.

In conclusion, the use of a mediastinal drain alone after esophageal cancer surgery was equally safe. Moreover, it could significantly reduce postoperative pain in patients. So, it may replace the closed chest drain in the clinic.

The present study had the following limitations. First, as this study was retrospective, potential selective bias could not be excluded. In future work, we plan to incorporate additional prospective randomized controlled trial designs and non-inferiority designs to further validate the findings of this study. Second, the sample size of this study may be small. Therefore, larger studies are still needed to further confirm whether the use of mediastinal drains alone is safe and effective after esophageal cancer surgery. Third, all groups in this study included some patients with neck anastomosis, which may have potentially influenced the results. Fourth, this study had not included the post-operative adjuvant treatment of patients in each group, and in the future, we will further follow up to explore the effect of different drainage methods on post-operative adjuvant treatment.

## Data Availability

The datasets used or analyzed during the current study are available from the corresponding author on reasonable request.
